# Neural Network-Enabled Process Flowsheet for Industrial Shot Peening

**DOI:** 10.3390/ma19010009

**Published:** 2025-12-19

**Authors:** Langdon Feltner, Paul Mort

**Affiliations:** School of Materials Engineering, Purdue University, 701 W Stadium Ave., West Lafayette, IN 47901, USA; pmort@purdue.edu

**Keywords:** shot peening, residual stress, flowsheet, digital twin, machine learning

## Abstract

This work presents a dynamic flowsheet model that predicts residual stress from shot peening. The peening medium is characterized by size and shape, and evolves dynamically with abrasion, fracture, classification, and replenishment. Because particle size and impact location vary stochastically, the resulting residual stress field is spatially heterogeneous. Residual stress fields are predicted in real time through a convolutional long short-term memory (ConvLSTM) neural network trained on finite element simulations, enabling fast, mechanistically grounded prediction of surface stress evolution under industrial shot peening conditions. We deploy the model in a pair of 10,000-cycle production peening case studies, demonstrating that media recharge strategy has a measurable effect on residual stress outcomes.

## 1. Introduction

### 1.1. Artificial Intelligence and Machine Learning for Material Design, Discovery, and Optimization

The development of advanced sensing technologies has opened new avenues for manufacturing process control, enabling dynamic systems that adapt to evolving process conditions. Digital twin frameworks have become central to the vision of Industry 4.0, with the potential to prevent the production of defective components in fatigue-critical applications [1].

Digital twins link process measurements, physics-based models, and control strategies in real time. Within this hierarchy, process flowsheets serve as the organizing structure: they capture the logical and physical sequence of process stages, enabling direct mapping between inputs, underlying deformation mechanisms, and performance outcomes.

Unlike implementations that rely primarily on sensor fusion and data-driven models, flowsheet-based twins emphasize mechanistic understanding of the process. They provide a scalable and interpretable foundation that can be augmented by statistical or physics-based corrections, ensuring that essential process–physics interactions are retained while remaining compatible with real-time monitoring and control [2].

The purpose of this paper is to introduce a reduced-order process flowsheet model of shot peening, a mechanical surface treatment widely used in the aerospace and automotive industries to extend the fatigue life of structural metallic components. A deep learning architecture combining convolutional neural networks with convolutional long short-term memory layers is used to predict residual stress fields from media recycle loop tracking. The model captures the stochastic, multiscale character of the process in a form suitable for integration into digital twin frameworks.

### 1.2. Shot Peening Overview

In shot peening, steel or ceramic particles (typically ∼1 mm in diameter) are propelled at high velocities (∼65 m/s) to bombard metallic components. This process plasticizes the near-surface region and induces compressive residual stresses, which enhance fatigue performance by increasing the applied tensile stress required to initiate or propagate cracks, while simultaneously modifying surface hardness and roughness.

The stochastic nature of impact locations and media morphology in shot peening necessitates a careful balance of process parameters to achieve optimal residual stress states and fatigue performance. Comprehensive reviews by John et al. and Xie et al. summarize conventional and advanced peening techniques, process parameters, and associated strengthening mechanisms across a wide range of alloys, while Bagherifard and Bagherifard & Guagliano highlight links between residual stress, microstructure, hardness, and fatigue performance in lightweight and nanocrystallized surfaces [3,4,5,6]. Świetlicki et al. [7] complement these works by providing a detailed review of shot peening methodologies and materials effects. Recent experimental and numerical studies further quantify the influence of peening velocity, coverage, and testing methodology on forming response, residual stress, and gear fatigue strength in industrial contexts [8,9,10]. Taken together, these results underscore that shot peening efficacy hinges on a nonlinear, material-specific interplay between blast pressure, media characteristics, and surface coverage.

### 1.3. Opportunities for Media Characterization

Industrial specifications for peening media are rooted in the traditional methods of sieve analysis (size) and qualitative visual inspection (shape), for example MIL-S-13165 originally issued in 1953 [11]. Since then, digital imaging technologies for size and shape characterization have advanced significantly and are now routine for many applications.

Steel shot peening media are commonly either cast or cut-wire, where cast media are made via gas atomization while cut-wire particles are cut from a drawn wire. Both types of media come in multiple grades (often defined by the roundness and hardness of the particles), but have significantly different archetypal shapes, microstructures, and therefore degradation mechanisms. Studies addressing media shape and breakage represent a gap in the literature this report seeks to lay out a reduced order approach for managing. Current work on the dynamic flowsheet employs dynamic image analysis (DIA) (Solidsizer, JM Canty, Lockport, NY, USA) with supplemental data analysis written in LabVIEW (National Instruments, Austin, TX, USA) to fit characteristic distributions for size and shape [12,13]. Reference Cut-Wire 32 (CW32) working mix media samples were provided by American Axle & Manufacturing (Detroit, MI, USA) and Toyo Seiko North America (South Bend, IN, USA). Shape distributions are used to calculate an effective radius of curvature on peening impact, while the size affects the mass (i.e., kinetic energy) associated with peening.

### 1.4. Challenges in Numerical Simulation

Finite element analysis is a common tool for modeling of shot peening, where representative volume element approaches simulate a set of particles comprising the average areal mass density impacting a subsection of the surface of a component in a random arrangement [14]. For example, Slimane et al. combine X-ray diffraction measurements with finite element simulations to characterize compressive residual stresses from shot peening under industrial conditions, illustrating both the capabilities and computational burden of high-fidelity models in practice [10]. While modern FEM solvers are flexible, including access to contact modules and numerous materials models, the fine mesh necessitated by nonlinear deformation behavior leads to severe computational overhead. In order to accurately capture the full distribution in residual stresses, RVE approaches should ideally scale with operational parameters and the media size. While RVE peening simulations with distributed media sizes have been used in the past [14], each simulation, especially those with sufficient area such that the average mass flux of impacts has reached relative stability, can take hours to run. In contrast, the typical peening process lasts a matter of seconds.

In a recent editorial publication for *The Shot Peener Magazine* [15], we developed a statistical approach for characterizing spatial uniformity in shot peening coverage by modeling impact events as a Poisson point process. This model captured the expected value, variance, and relative standard deviation in impact counts over finite surface regions, incorporating parameters such as media mass flux ($\dot{m}$), part area ($A_{part}$), peening duration ($t_{c}$), and a lognormal media size distribution. However, that treatment focused solely on the statistics of impact placement and did not establish a link between impact field variability and the resulting residual stress fields.

The present work aims to bridge that gap by introducing a computational framework that couples transient process conditions with evolving media morphology and spatial stress predictions. Specifically, we develop:

(1) A reduced-order flowsheet model to track media size and shape evolution under wear, breakage, and replenishment cycles.
(2) A stochastic coverage simulator that maps operational parameters to spatially distributed impact conditions.
(3) A convolutional long short-term memory surrogate trained on high-fidelity FEM data to rapidly predict local residual stresses from sampled impact fields.

Together, these components form a high-speed process flowsheet with potential extensions to a digital twin architecture for process-aware, data-driven prediction of shot peening outcomes.

## 2. Materials and Methods

### 2.1. Flowsheet Architecture

The process flowsheet tracks the circulation of peening media as a dynamic working mix composed of three characteristic modes: (1) as-manufactured, (2) conditioned, and (3) worn. As media are cycled through repeated impacts, they undergo morphological degradation via fracture, abrasion, and plastic deformation. When particles reach a state of excessive wear, they are removed from the system by a classifier and discarded as debris. To maintain system equilibrium, discarded media are replenished with fresh (as-manufactured) particles based on a mass-balance threshold, resulting in a continuous renewal process that stabilizes the working mix distribution over time.

**Figure 1:**
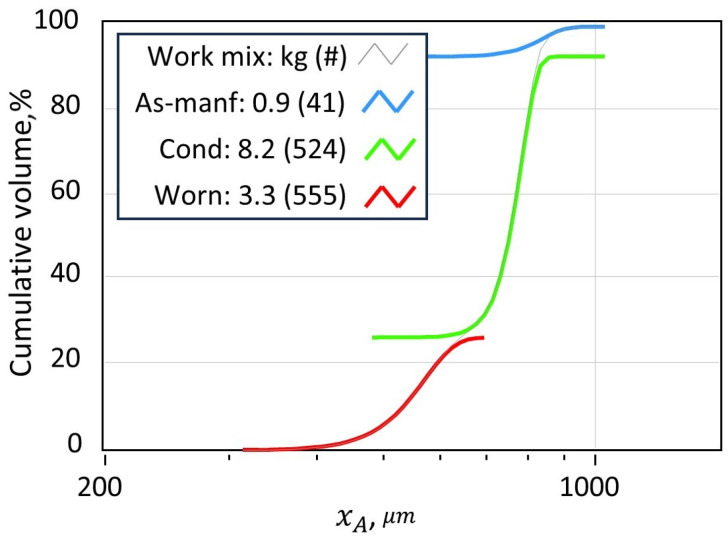
Snapshot of the flowsheet simulation showing a mixture of three modes, each with a mass (kg) and average number of impact cycles (#).

Each mode is further characterized by distinct size and shape distributions, quantified using DIA and parameterized using stretched exponential fits. As shown in Figure 1 and summarized in Table 1, the size of media decreases monotonically with wear, while the tightest distributions occur in the conditioned mode. Shape anisotropy, described by aspect ratio (AR), increases in uniformity during the transition from as-manufactured to conditioned media, but becomes broader again in the worn mode due to fracture and distortion.

The working mix model captures this evolving distribution as a three-mode mixture, with the relative contribution of each mode governed by the age of particles in the system. Transitions between modes are described using empirical wear parameters, which may be directly measured using a cyclical-impact apparatus (Ervin Tester, Ervin Inc., Adrian, MI, USA) or inferred through multimodal decomposition of dynamic image analysis (DIA) data from working-mix samples [13]. Each mode is modeled using a Weibull lifecycle function, where the cumulative distribution of transfers, $x_{T}$, is given in terms of the characteristic number of impact cycles $x^{*}$ and a stretching exponent *m*:

$$x_{T}=1-\exp\left(-{\left(\frac{x}{x^{*}}\right)}^{m}\right).$$

This formulation enables probabilistic modeling of inter-mode transfer, where the likelihood of a particle transitioning out of its current mode increases with cumulative impact exposure.

For example, fresh media introduced into the system begin in the as-manufactured mode. According to the wear parameters in Table 2, these particles transition to the conditioned mode with a relatively low characteristic impact count ($x^{*}=1000$) and a low stretching exponent (m=1), indicating rapid conditioning of most of the media with a diffuse tail. Once in the conditioned mode, media are more resilient, with a higher durability ($x^{*}=4000$). The stretching exponent (m=4) delays the conditioned → worn transition, with the effect that conditioned particles tend to remain stable over many impacts before transitioning into the worn state. Worn particles degrade with an intermediate profile before exiting the system as debris, with $x^{*}=1600$ and m=2. Overall, the wear parameters define a cycle of degradation, where particles are continuously added, reshaped, and ultimately removed, with the recharge strategy maintaining a dynamic equilibrium across the three modes. This evolving distribution directly influences the impact energy spectrum delivered to the peened surface.

#### 2.1.1. Work Mix Dynamics

The mix of modes is dynamic and depends on the media recharge criteria. The current version of the flowsheet must have a heel of media in a recycle bin. When the mass in that bin drops below a threshold value, a recharge of the as-manufactured media occurs. If the recharge mass is small relative to the total working mix, i.e., frequent and small recharges, the working mix is relatively stable with dampened fluctuations occurring primarily between the conditioned and worn modes. If the recharge is large and less frequent, the working mix will fluctuate substantially among all three modes. Note, the current flowsheet assumes stable operation of the classifier, i.e., there are no parameters for screening efficiency in the current model.

#### 2.1.2. Archetypal Models for Peening Media

**Figure 2:**
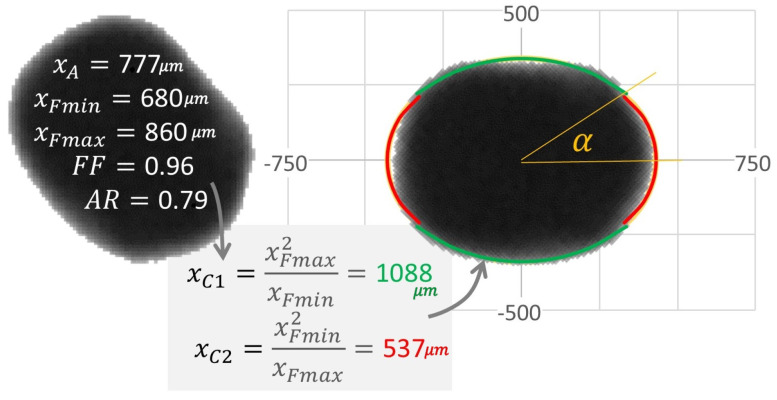
Translation from image analysis data to distributed characteristics ($x_{A}$, AR) to simplified elliptical archetype having contact curvature. Example is a 2D DIA image of a worn CW32 shot. Axis units in μm.

Shape archetypes were used to translate measured shape distributions into effective curvature of peening impacts. Archetypes describe the peening media in terms of their method of manufacture (e.g., cast versus cut wire steel) and level of conditioning. The current work employs an elliptical archetype model for conditioned cut wire, having effective curvature defined by major and minor axes, shown in Figure 2. Note that unconditioned cut wire has sharper curvature at cut-corners, yet these features are relatively short-lived in a steady-state peening process. While quantitative descriptions of detailed curvature features have been enabled in image analysis [16], the simpler elliptical archetype shape model was used for the purpose of flowsheet demonstration [17].

Peening media size and shape effects are selected using a two-step process: (1) random selection of a peening media mass based on the number distribution of the measured area-equivalent size, $x_{A}=\sqrt{4A/\pi }$, and (2) random selection of an elliptical curvature based on an archetypal shape model. In this example, the measured aspect ratio distribution, $AR=x_{F\min}/x_{F\max}$, is used to estimate the curvature of an elliptical archetype. Empirically, we found $x_{A}$ and AR to be uncorrelated within each mode, allowing independent sampling.

To estimate contact curvature, we approximate each particle as an ellipse with semi-major axis *a* and semi-minor axis *b*, related to the area-equivalent size and aspect ratio by:

$$x_{A}=\sqrt{ab},\;AR=\frac{b}{a},\;\Rightarrow \;a=\frac{x_{A}}{AR^{1/2}},\;b=x_{A}\cdot AR^{1/2}.$$

The principal radii of curvature at the tips of the ellipse are given by:

$$R_{a}=\frac{b^{2}}{a}=x_{A}\cdot AR^{1.5},\;R_{b}=\frac{a^{2}}{b}=\frac{x_{A}}{AR^{1.5}},$$

which define the elliptical curvatures used in the model:

$$x_{C1}=\frac{x_{A}}{AR^{1.5}},\;x_{C2}=x_{A}\cdot AR^{1.5},$$

corresponding to the sharper (minor-axis tip) and flatter (major-axis tip) regions of the particle, respectively.

The relative probability of a peening contact occurring at $x_{C1}$ versus $x_{C2}$ depends on the ray length from the center of mass to the point of curvature:

$$P_{1}=\frac{x_{F\min}}{x_{F\min}+x_{F\max}},\;P_{2}=\frac{x_{F\max}}{x_{F\min}+x_{F\max}}.$$

**Figure 3:**
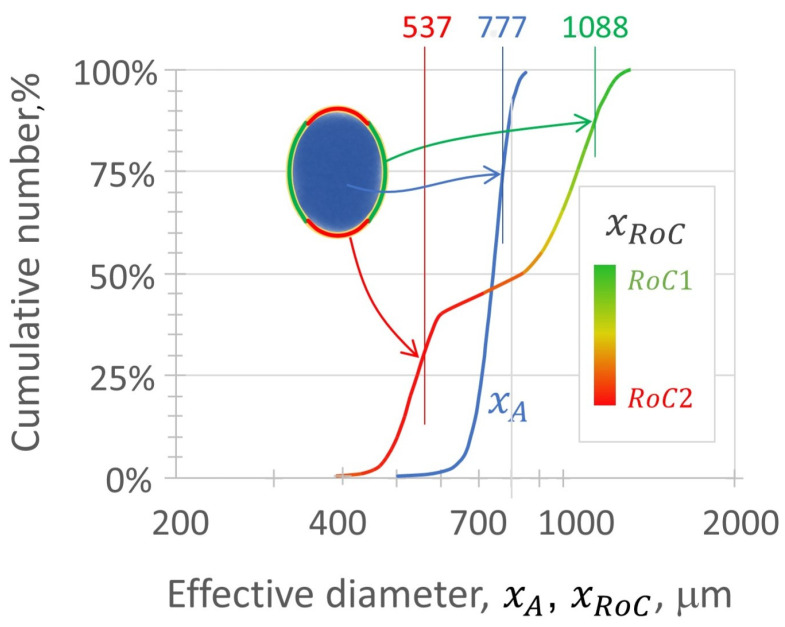
Effective contact curvature distribution obtained by applying the elliptical shape archetype to CW32 peening media, $x_{C}$, compared with the area-equivalent distribution, $x_{A}$.

The selection of $x_{C1}$ versus $x_{C2}$ is made by an additional random number call based on these probabilities. Overall, the flowsheet integrates size and shape distributions using a Monte Carlo statistical approach involving three random choices: (1) selection of $x_{A}$, (2) selection of AR, and (3) selection of $x_{C}$ from the directional curvatures. This provides a distribution of discrete peening events, each having an impact energy and contact curvature. When considered as contacts, the curvature model has the effect of broadening the media distribution (Figure 3).

#### 2.1.3. Effective Peening Impact

**Figure 4:**
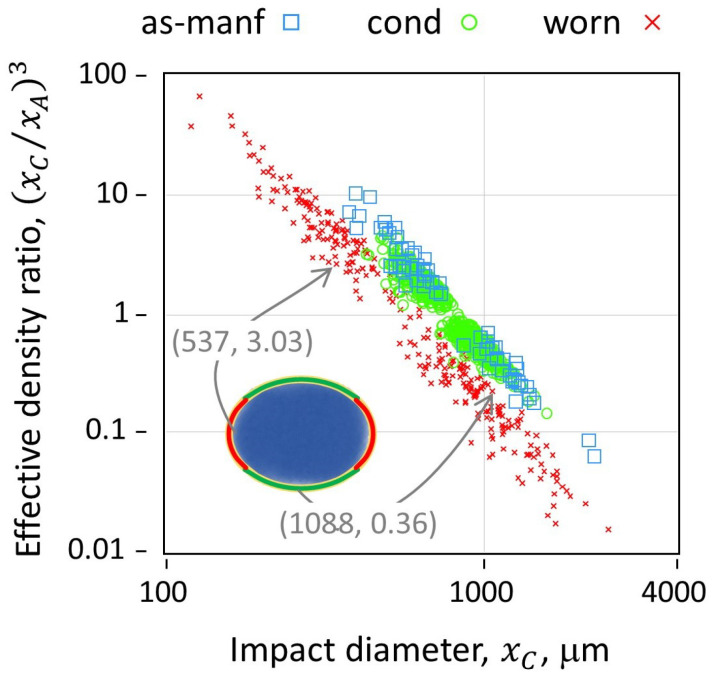
Effective density ratio used to maintain the effective kinetic energy (i.e., mass) of media impacting with adjusted curvature (i.e., shape), using same snapshot as Figure 1. Inset is an example of the elliptical shape archetype.

Each media-substrate impact is modeled as an effective impact between a spherical shot particle having a radius that is equal to the selected radius of curvature, yet having a mass that is consistent with the actual shot size. The actual mass is based on the material density, e.g., 7.8 g/mL for CW32 media, multiplied by the volume of the shot particle obtained from its projected area, *A*, measured by DIA, $V=\left(4/3\right)\cdot A^{1.5}/\sqrt{\pi }$. To maintain this mass with a sphere that was adjusted to a different radius of curvature, an effective density ratio is applied, $DR=\rho_{eff}/\rho_{shot}={\left(x_{A}/x_{C}\right)}^{3}$. When $x_{C}>x_{A}$, the effective density ratio < 1; when $x_{C}<x_{A}$, the effective density ratio > 1 (Figure 4). This suggests a broadening of the impact stress distribution, with large-curvature impacts having relatively broader contact area and lower impact stress compared to small-curvature impacts.

#### 2.1.4. Peening Coverage

The flowsheet simulation generates periodic snapshots of peening coverage based on the prescribed media mass flow rate, treated surface area, and a defined coverage time interval. Impacts contributing to the average areal mass density are sampled over a representative surface element, with impact locations modeled using randomized (x,y) coordinates.

**Figure 5:**
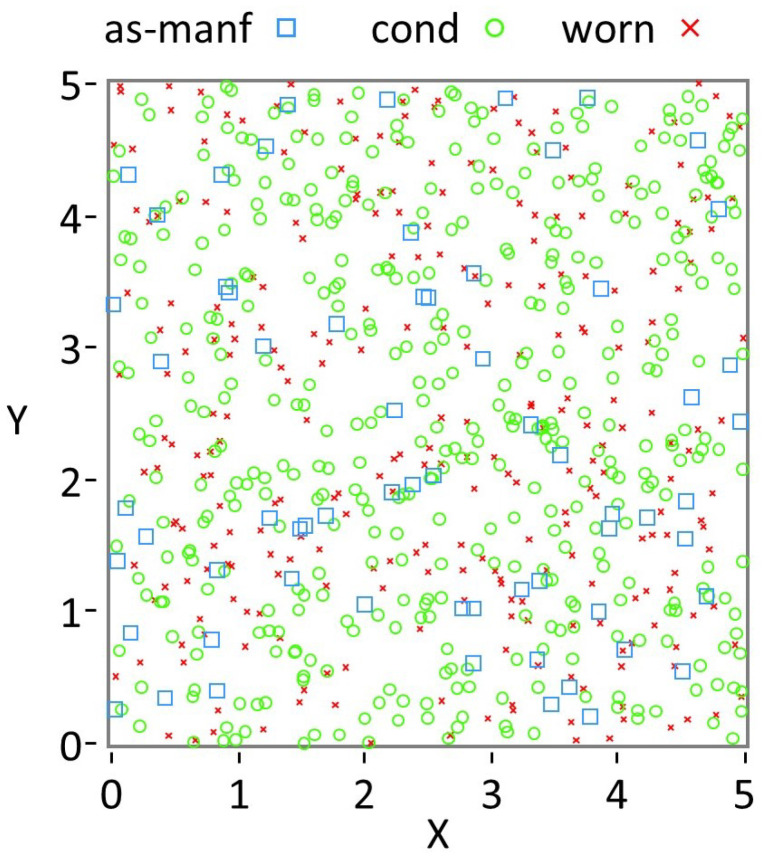
Coverage map of a representative volume element by media type, using same snapshot as Figure 1.

The coverage map is displayed as point contacts by mode within the work mix distribution (Figure 5). The data shown in Figure 4 provide initial conditions for more detailed residual stress calculations via Finite Element Modeling (FEM) and reductions thereof, essentially correlating the distribution of paired input parameters ($x_{A},x_{C},DR$) to a resultant stress field induced by the random coverage (Figure 5) of said impacts.

### 2.2. FEM-Based Convolutional Neural Network

#### 2.2.1. RVE Finite Element Analysis

Snapshots of peening coverage generated by the flowsheet (Figure 5) were used as initial conditions for finite element simulations. These coverage maps, defined by sampled impact positions and media descriptors ($x_{A},x_{C},DR$), provide the basis for correlating stochastic impact fields with resultant residual stress states and for constructing the training dataset for the ConvLSTM surrogate.

Each FEM simulation drew coverage maps from the flowsheet while varying mass flow rate and impact velocity within the ranges given in Table 3, with fixed constants summarized in Table 4. The full factorial design yielded 9 simulations. Radii of curvature were assigned from elliptical archetypes based on measured aspect ratios. Shots were treated as rigid, impacts were temporally staggered, and a node-to-surface contact model without friction was applied. For each simulation, residual stress fields were recorded after the series of impacts. These stored fields, paired with their corresponding impact histories from the flowsheet, form the basis for the patch-level dataset used to train and evaluate the ConvLSTM surrogate.

Johnson–Cook isotropic hardening was used to model the elasto-plastic response of the SAE 1070 Almen strip material, as described by Ghanbari et al. [18] (Table 5). Thermal effects were not simulated; peening was treated purely as a cold-working process.

ABAQUS Explicit 2019 [19] was used to run the peening simulations. A rectilinear mesh of C3D8R elements made up the bulk of the RVE. The simulated RVE was 5 mm × 5 mm × 2 mm in size, with a layer of infinite (CIN3D8) elements at its boundary and base. Grid independence on the basis of total strain energy was observed across all combinations of impact kinetics with a surface element size of 20 μm × 20 μm × 20 μm, in line with guidance by Wang et al. [20], who observed grid independence with a surface element size of 1/10-th the dimple diameter. For computational efficiency, a biased mesh was employed such that elements farther from the impact surface were larger, with a maximum element size of 20 μm × 20 μm × 100 μm at the base of the substrate.

For this report, we focus on the mean in-plane residual stress, defined as

$$\sigma_{\mathrm{mean}}=\frac{1}{2}\left(\sigma_{11}+\sigma_{22}\right),$$

because it is translationally and rotationally invariant relative to the measurement plane and directly related to the pressure holding a surface crack closed. Future work may also consider the normal Cauchy stress in the *X* or 11-direction ($\sigma_{11}$), given its direct analogy to stresses measured with X-ray diffraction [21,22], which remains a common quality control metric in industrial shot peening practice. In this work we restrict attention to normal impacts, where the stress state is approximately isotropic within the surface plane; however, off-angle cases may introduce anisotropy into the residual stress field and necessitate directionally dependent descriptors.

#### 2.2.2. LSTM Convolutional Neural Network FEM Surrogate

In our previous work [23], we used spectral correction methods to describe residual stress field structure based on reduced-order micromechanical solutions. That approach did not account for impact order, instead relying on linear superposition of impacts. At low impact densities, where overlaps are rare, the model successfully reproduced both the pointwise stress field and its spatial correlation structure. At high impact densities, however, the reduced-order model simplified to a stochastic field with the same mean, variance, and correlation structure as the FEM predictions, but without the ability to resolve pointwise details. To address this limitation, the present work treats the evolution of stresses and surface topography in the shot peening system as a history-dependent process.

We developed an impact-order–aware ConvLSTM surrogate model trained on a set of representative finite element simulations, enabling fast prediction of residual stress fields under arbitrary peening conditions. Training inputs were constructed as spatial energy maps using an Eshelby inclusion analogy, as explored in our recent work [23]. Each impacting particle was represented by a 2D projection whose footprint was parameterized by the effective contact radius derived from the selected contact curvature rather than the full geometric projection. Within this footprint, the particle’s kinetic energy was distributed uniformly; points outside received zero contribution. Thus, each impact was encoded as a localized scalar energy inclusion capturing the combined effects of particle size, shape, and velocity:

$$E_{i}\left(x,y\right)=\left\{\begin{array}{cc} \frac{1}{\pi r_{i}^{2}}\left(\frac{1}{2}m_{i}v_{i}^{2}\right), & \mathrm{if}\;{\left(x-x_{i}\right)}^{2}+{\left(y-y_{i}\right)}^{2}\leq r_{i}^{2},\\ 0, & \mathrm{otherwise}, \end{array}\right.$$

where $m_{i}$ and $v_{i}$ are the mass and velocity of the *i*th particle, and $r_{i}$ is the effective contact radius used for input construction. For each stored FEM snapshot, we reconstructed the preceding impact history from the flowsheet and rasterized the corresponding sequence of energy inclusions onto a fixed grid. Each dataset sample consists of a temporally ordered impact sequence paired with the resulting local stress response.

FEM-predicted stress fields were decomposed into local prediction windows to provide training targets for the surrogate model. Each target was defined as a 16×16 pixel patch of the in-plane residual stress field, corresponding to a neighborhood of approximately $320\times 320\;\mu m^{2}$ at the FEM mesh resolution. Predicting continuous patches simultaneously, rather than individual points, improved the model’s ability to reproduce coherent structures associated with surface plasticity and reduced the numerical model’s tendency to minimize variance across the field. This window size was chosen to balance spatial resolution with computational efficiency, while remaining large enough to capture the stress gradients and local interactions among multiple adjacent impacts. For each RVE and coverage snapshot, the full $5\times 5\;{\mathrm{mm}}^{2}$ surface was tiled into overlapping 16×16 patches using a fixed stride, and patches near free boundaries were excluded to avoid edge artifacts. Patches from all simulations and coverage levels were then pooled, randomly shuffled, and used to assemble the final dataset.

The corresponding model input was an ordered sequence of impact “frames,” each of size 36×36 pixels, encoding the spatial distribution of particle energy deposition across successive timesteps. The larger input frame ensured that the receptive field encompassed the surrounding impact environment beyond the target window, allowing the network to capture influential impacts just outside the prediction region. Each input–output pair therefore consists of a sequence of *T* impact-energy frames of size 36×36 and a corresponding 16×16 residual stress patch, where *T* is the number of impacts that intersect the region of interest.

Our network was implemented in TensorFlow [24]. The architecture, summarized in Table 6, was designed to balance temporal–spatial modeling capacity with computational efficiency. A per-frame encoder reduced each 36×36 impact frame to progressively compressed feature maps (18×18 and 9×9), using gelu activations to enhance convergence stability without explicit normalization. These encoded sequences were passed to stacked ConvLSTM layers, which captured both intra-frame spatial correlations and inter-frame temporal dependencies. The decoder then upsampled the 9×9 latent representation back to a 16×16 output window, using bilinear interpolation followed by convolutional refinement to reconstruct fine-scale detail. A final 1×1 convolution produced the predicted residual stress field. This encoder–ConvLSTM–decoder formulation enabled mappings from evolving impact fields to localized stress responses without the computational burden of full-field predictions.

We employed a custom loss function that combined the mean squared error (MSE) of the $16\times 16\;{\mathrm{pixel}}^{2}$ stress patches with the difference in variance between the ML predictions and the FEM targets. This formulation rewarded point-wise accuracy while penalizing the model’s tendency to underestimate field-level variance. Both components of the loss are dimensionally consistent (${\mathrm{GPa}}^{2}$). The dataset was partitioned into a 75:25 training–test split. Patches were assigned to the training or test set at the sample level, such that each input–output pair was used exclusively for training or evaluation. Training used the AdamW optimizer with an initial learning rate of $6\times 10^{-4}$, progressively reduced to $1.5\times 10^{-4}$ as losses plateaued. After 42 epochs, the model achieved a final MSE of $0.0443\;{\mathrm{GPa}}^{2}$ and an average variance difference of $0.0113\;{\mathrm{GPa}}^{2}$ on the test set.

## 3. Results and Discussion

### 3.1. Patch-Level Accuracy

**Figure 6:**
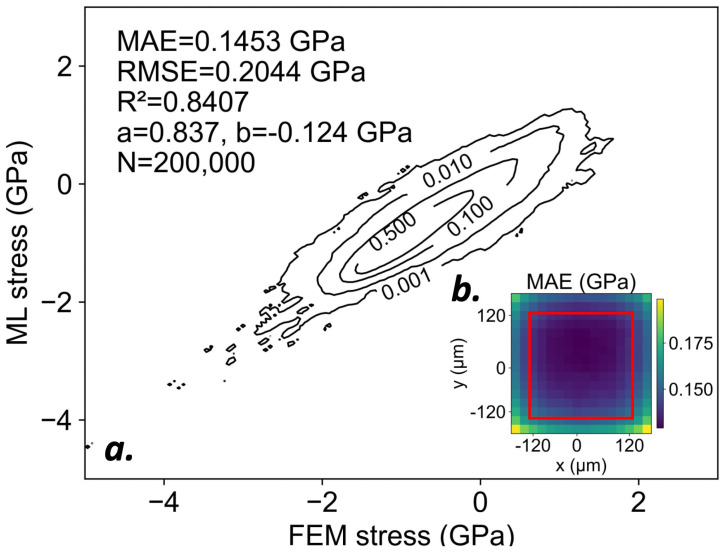
(**a**) Contour parity plot comparing FEM-computed and ML-predicted stresses at the pixel level. (**b**) Per-pixel mean absolute error (MAE) evaluated across the prediction window. Central 12×12 region marked in red.

Figure 6a summarizes parity statistics for 200,000 randomly sampled pixels from the test set. Relative to an overall mean stress magnitude of 0.818GPa, the model achieved a mean absolute error (MAE) of 0.1453GPa. The fitted regression slope (a=0.837) and intercept (b=−0.124GPa) indicate a tendency to overpredict the magnitude of low-stress regions and underpredict the magnitude of high stress regions. Nevertheless, the model generalizes well to the test data and successfully reproduces FEM-predicted stress concentrations with magnitude greater than 2GPa, which occur in 0.1% of sampled pixels.

Figure 6b further examines performance at the per-pixel level. Although all pixels exhibited a mean absolute error below 0.200GPa, those located on the edges of the prediction window performed worse than those at the center. Overall, there was little variation in accuracy within the central 12×12 region of the prediction window.

### 3.2. Structural Accuracy and Generalization Across Conditions

**Figure 7:**
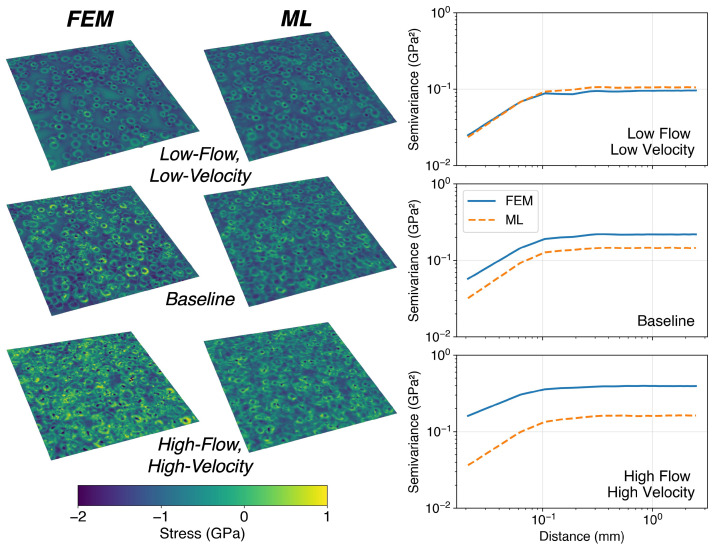
Comparison of FEM and ML-predicted stress fields tiled from 12×12 patches to form $5\times 5\;{\mathrm{mm}}^{2}$ RVEs. (**Left**): Representative stress fields for baseline and extreme cases. (**Right**): Corresponding variograms showing semi-variance as a function of separation distance.

We used the central 12×12 region of the prediction window to tile $5\times 5\;{\mathrm{mm}}^{2}$ RVE stress fields and assess the model’s ability to reproduce correlation structures across scales larger than a single tile. Figure 7 compares stress fields for the baseline case and two extreme conditions of the full factorial design (low flow rate with low velocity, and high flow rate with high velocity). While portions of these fields contributed patches during training, the model never observed complete fields; tiling therefore provides a test of generalization beyond patch-level memorization. These visualizations demonstrate that the model captures field-level organization under contrasting process conditions.

Variograms on the right of Figure 7 depict the expected squared difference (semi-variance) of stress values separated by a given distance. The plasticized region beneath an impact forms a coherent structure within the stress field, where nearby elements exhibit similar stresses. Consequently, semi-variance increases with separation distance until reaching a plateau that reflects the characteristic extent of the plasticized zone. Beyond this range, stress fluctuations appear largely stochastic and spatial organization decays. The horizontal asymptote approached at large separations corresponds to the variance of the stress field.

Table 7 directly compares field-level means and standard deviations across the three tested operating conditions. Increasing flow rate and impact velocity intensifies surface plasticity and penetration depth, which in turn decreases the mean compressive stress magnitude at the surface while increasing variability. Similar phenomena have been reported separately by Klump et al. [25] and Wang et al. [26].

The ML model best reproduces the low-flow rate case, accurately capturing the mean stress, variance scaling, and correlation structure. In this regime, impact dimples form a network of connected tensile rings due to localized upheaval of the surface. The relatively sparse coverage allows these features to remain intact, and they are well reproduced by the ML model. Accounting for impact sequence is critical here, since the most recent impact deforms and reorganizes the surface beneath.

In the high-flow rate case, overlapping impacts severely deform portions of the surface, pushing the limits of both FEM and ML predictions. The network of impact dimples is largely diminished, and—as reflected by the shallower slope of the variograms in the correlated region—both fields begin to resemble random stress distributions.

The baseline case represents an intermediate condition, with impact structures that are partially preserved but also subject to overlap and smoothing. The ML model closely matches the FEM-predicted mean value (−0.824 GPa vs. −0.853 GPa). While the ML model tends to underestimate variance at higher impact coverages, it retains 82% of the FEM-predicted standard deviation in the baseline condition.

Overall, the ML model consistently preserves correlation length scales, indicating that it captures the essential spatial organization of the stress field. Combined with the strong parity performance against FEM data, this fidelity is sufficient for the model to serve as a reduced-order process monitoring tool. In particular, it enables exploration of stress field dynamics under baseline conditions, where cycles of media recharge and breakdown drive temporal evolution of surface morphology and residual stress distributions.

### 3.3. Temporal Dynamics

**Figure 8:**
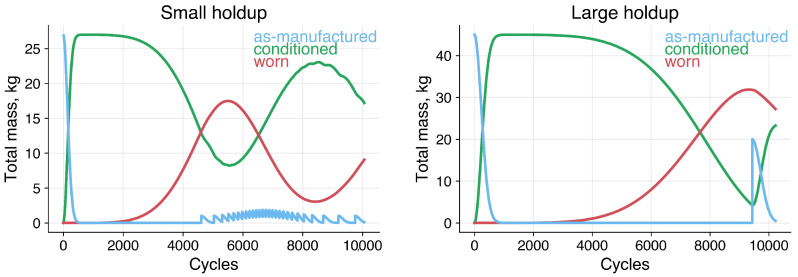
Media recharge strategies for small (20 kg heel, 1 kg recharge) and large (30 kg heel, 20 kg recharge) holdup cases.

Consider two media recharge strategies shown in Figure 8. In both cases, approximately 5 kg of media circulate through the blast loop each cycle, while the remainder resides in the holdup hopper. In the small-holdup case, the process is initialized with 26 kg of media in total. The holdup is maintained at a 20 kg threshold, with 1 kg of new media added whenever the holdup mass falls below this limit, resulting in frequent, gradual renewals after an initial conditioning period. In the large-holdup case, the process begins with 45 kg of as-manufactured media in total. Here, the holdup is maintained at 30 kg, and 20 kg are added once that threshold is reached, producing less frequent but more substantial renewal events.

**Figure 9:**
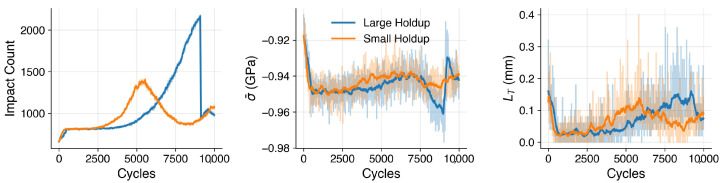
Evolution of average stress $\left(\bar{\sigma }\right)$, maximum tensile path length $\left(L_{T}\right)$, and impact count over 10,000 cycles for small and large holdup cases. Transparent traces show raw simulation outputs recorded every 10 cycles, while dark lines indicate 25-point rolling averages for clarity.

We simulated 10,000 peening cycles, taking RVE impact map samples every 10 cycles and passing them through the ML model. Figure 9 summarizes trends in average stress $\left(\bar{\sigma }\right)$, maximum tensile path length $\left(L_{T}\right)$, and impact count. The tensile path length, $L_{T}$, quantifies the spatial continuity of tensile regions within the predicted surface stress field. It is computed using an 8-connected flood-fill search [27] with periodic boundary conditions, which systematically traces the longest contiguous cluster of tensile elements (σ>0) across the surface. This approach captures not only the magnitude but also the geometric persistence of tensile features—analogous to flaw-like regions that can facilitate crack initiation or locally reduce compressive effectiveness. Stable, confined tensile paths reflect uniform and well-contained plastic deformation, whereas sporadic increases in $L_{T}$ mark rare events in which extended tensile bands emerge, signaling potential degradation in peening quality and increased susceptibility to crack growth.

At startup, both strategies are identical: as-manufactured media transition into the more resilient conditioned state, accompanied by a ∼30 MPa shift toward more compressive surface stress. Higher impact counts and smaller, more uniform dimples from the reduced particle size shorten the maximum tensile path length $\left(L_{T}\right)$, indicating a more homogeneous and spatially confined stress field.

The smaller media mass in the small holdup case accelerates the onset of the worn state—around 2000 cycles versus 3000 in the large holdup case. Impact counts continue to rise, but increased shape anisotropy in the worn mode promotes the formation of extended tensile features, causing the maximum tensile path length $\left(L_{T}\right)$ to increase even as $\bar{\sigma }$ remains relatively stable.

In the small holdup trial, the worn mode dominates at ~4500 cycles, coinciding with the start of frequent recharges. Each recharge of as-manufactured media replenishes the conditioned mode, establishing a relatively stable renewal cycle and reducing both impact count and $L_{T}$.

In the large-holdup case, worn media is predominant prior to recharge. Impact counts more than double relative to the early conditioned state, and $L_{T}$ rises accordingly. Upon replenishment, 20 kg of fresh media is added to 30 kg of worn, sharply reducing the impact count. For approximately 200 cycles, the combination of worn and as-manufactured particles—both exhibiting high shape anisotropy—drives $L_{T}$ to a maximum and induces a ∼25 MPa shift toward less compressive $\bar{\sigma }$. In some instances, $L_{T}$ values exceeding 0.3 mm were observed—on the order of a particle radius. Such events represent measurable drops in peening process quality, increasing the likelihood of flaw formation and localized tensile persistence at the surface.

## 4. Conclusions

This work introduces a reduced-order process flowsheet for industrial shot peening that couples a three-mode media degradation model with a convolutional long short-term memory (ConvLSTM) surrogate for residual stress prediction. The flowsheet tracks the joint evolution of media size, shape, and impact statistics under different recharge strategies, while the ConvLSTM model provides real-time predictions of high-resolution residual stress fields based on finite element (FEM) training data. The FEM reference fields are computed using a Johnson–Cook material model calibrated against experimental data for SAE 1070 Almen strips, providing experimental traceability for the numerical benchmark.

Across a full factorial of operating conditions, the ConvLSTM surrogate achieves a patch-level mean absolute error of approximately 0.145GPa relative to an average stress magnitude of 0.818GPa, successfully reproducing rare stress concentrations exceeding 2GPa. Tiled predictions preserve FEM-predicted field statistics, including mean stress, variance, and correlation length scales under low-flow, baseline, and high-flow conditions.

When embedded in the flowsheet, the surrogate enables production peening simulations for contrasting media recharge strategies, revealing distinct trajectories in near-surface mean stress, impact count, and maximum tensile path length $L_{T}$. These results demonstrate that media management exerts a direct influence not only on the mean residual stress state but also on the spatial heterogeneity and connectivity of tensile features at the surface.

These scenarios serve as a proof of concept for how the model can capture the coupled evolution of media states, impact statistics, and stress variability under different operational strategies. Beyond reproducing qualitative trends, the framework demonstrates potential as a tool for evaluating alternative recharge policies and linking them directly to surface integrity outcomes.

The high-resolution stress fields produced by the ML model provide a basis for alternative quality control metrics beyond mean-field diffraction-based experiments. In particular, future work investigating relative variability and characteristic correlation length effects on crack propagation dynamics could seek to establish quantitative relationships between stress field heterogeneity and fatigue performance. Such relationships would enable the model to serve not only as a predictive tool but as a foundation for performance-based process qualification. A dedicated experimental study combining in-process media characterization with depth-resolved residual stress measurements (e.g., X-ray diffraction or layer-removal methods) under matched peening conditions will be an important next step to validate both the FEM model and the ConvLSTM surrogate against physical measurements.

When coupled with in-process sensing of media condition and impact statistics, this framework could bridge model prediction and experiment to form the foundation of a full digital twin for shot peening. A key next step lies in informing media wear and velocity dynamics through direct measurement. Dynamic image analysis (DIA) can provide real-time distributions of particle size and shape to constrain media evolution, while high-speed velocimetry can characterize the temporal dynamics of impact energy. Integrating these data streams into the flowsheet would enable a continuously calibrated model capable of adaptive process control and predictive maintenance in industrial operation.

## Figures and Tables

**Table 1:** Example of size and shape parameters obtained by stretched-exponential fitting of dynamic image analysis data for CW32 media.

Mode	Size: $x_{A}$		Shape: *AR*	
	$d_{g}^{*}$	n	${\mathrm{AR}}^{*}$	n
As-Manufactured	850	15	0.80	10
Conditioned	790	20	0.90	20
Worn	575	10	0.75	6

**Table 2:** Example of wear parameters used in the modal transfer function, conditioned CW32 media.

Transfer Function	$x^{*}$	*m*
Manf → Cond	1000	1
Cond → Worn	4000	4
Worn → Debris	1600	2

**Table 3:** Range of operational parameters used to generate impact conditions for RVE simulations. The mass flow rate governs cumulative areal mass density, while the impact velocity influences particle kinetic energy.

Parameter	Lower Bound	Mean Value	Upper Bound
Mass Flow Rate (kg/s)	0.04725	0.0945	0.189
Impact Velocity (m/s)	45	65	85

**Table 4:** Fixed operational constants used in the peening simulation framework. $A_{\mathrm{part}}$ denotes the peened surface area, $t_{c}$ is the duration of a full peening cycle, and θ is the angle of incidence between the particles and RVE.

Parameter	Value
$A_{\mathrm{part}}\;\left({\mathrm{mm}}^{2}\right)$	49,100
$t_{c}\;\left(s\right)$	30
θ (∘)	90

**Table 5:** Elasto-plastic material properties for Johnson–Cook hardening model of SAE 1070 Almen strip steel [18]. *E* and ν denote Young’s modulus and Poisson’s ratio respectively.

Param.	*E* (MPa)	ν	*A* (MPa)	*B* (MPa)	*n*	*C*
Value	210,000	0.31	1408	600	0.234	0.0134
Param.	*m*	${\dot{\varepsilon }}_{0}$	$T_{0}$ (K)	$T_{M}$ (K)		
Value	1	1	298	1793		

**Table 6:** Neural network architecture mapping T×36×36×1 input frames to a 16×16×1 stress patch.

Layer	Output Shape	Act.	Params
Input	(T,36,36,1)	–	0
TD Conv2D (3 × 3, 16, s2)	(T,18,18,16)	gelu	160
TD Conv2D (3 × 3, 32, s2)	(T,9,9,32)	gelu	4640
ConvLSTM2D (48, 3 × 3, seq)	(T,9,9,48)	tanh/sig.	138,432
ConvLSTM2D (64, 3 × 3)	(9,9,64)	tanh/sig.	258,304
Conv2D (3 × 3, 64)	(9,9,64)	gelu	36,928
UpSample (2×)	(18,18,64)	–	0
Conv2D (3 × 3, 64)	(18,18,64)	gelu	36,928
Conv2D (3 × 3, 32)	(18,18,32)	gelu	18,464
Conv2D (3 × 3, 32, valid)	(16,16,32)	gelu	9248
Conv2D (1 × 1, 1)	(16,16,1)	linear	33
Total	–	–	503,137

**Table 7:** Mean and standard deviation (STD) of ML-predicted and FEM-computed residual stresses.

Condition	Mean [GPa]		STD [GPa]	
	**ML**	**FEM**	**ML**	**FEM**
Low flow/low velocity	−0.953	−0.964	0.325	0.309
Baseline	−0.824	−0.853	0.381	0.466
High flow/high velocity	−0.622	−0.509	0.403	0.628

## Data Availability

The simulation codes created in this study are openly available on GitHub at: https://github.com/feltner515/ML_Flowsheet, (accessed on 15 October 2025). Due to the large size of the raw finite element and machine learning datasets, these data are not publicly archived but are available from the corresponding author upon reasonable request.

## Abbreviations

The following abbreviations are used in this manuscript:

AI	Artificial intelligence
ML	Machine learning
CNN	Convolutional neural network
LSTM	Long short-term memory
ConvLSTM	Convolutional long short-term memory
FEM	Finite element modeling
RVE	Representative volume element
DIA	Dynamic image analysis
AR	Aspect ratio
DR	Density ratio
MAE	Mean absolute error
MSE	Mean squared error
RSD	Relative standard deviation
SAE	Society of Automotive Engineers
CW32	Cut-wire 32 (shot media grade)
GELU	Gaussian error linear unit (activation function)
AdamW	Adam optimizer with weight decay

## References

[1] Jones D., Snider C., Nassehi A., Yon J., Hicks B. (2020). Characterising the Digital Twin: A systematic literature review. CIRP J. Manuf. Sci. Technol..

[2] Palotai B., Kis G., Abonyi J., Bárkányi Á. (2025). Surrogate-based flowsheet model maintenance for Digital Twins. Digit. Chem. Eng..

[3] John M., Kalvala P.R., Misra M., Menezes P.L. (2021). Peening techniques for surface modification: Processes, properties, and applications. Materials.

[4] Xie X., Zhang L., Zhu L., Li Y., Hong T., Yang W., Shan X. (2023). State of the art and perspectives on surface-strengthening process and associated mechanisms by shot peening. Coatings.

[5] Bagherifard S. (2019). Enhancing the structural performance of lightweight metals by shot peening. Adv. Eng. Mater..

[6] Bagherifard S., Guagliano M. (2009). Review of shot peening processes to obtain nanocrystalline surfaces in metal alloys. Surf. Eng..

[7] Świetlicki A., Szala M., Walczak M. (2022). Effects of shot peening and cavitation peening on properties of surface layer of metallic materials—A short review. Materials.

[8] Wu J., Chen K., Zhang P., Zhu J., Liu G., Wei P., Liu H. (2025). Effects of peening velocity and coverage on peen forming. Proc. Inst. Mech. Eng. Part E J. Process Mech. Eng..

[9] Chen D., Gong L., Wu J., Qin G., Liu H. (2025). A study on the key influencing factors of gear fatigue strength testing based on the Locati method. Measurement.

[10] Slimane A., Bahram K., Chaib M., Dahmane S., Slimane S., Ait Kaci D., Ziadi A., Bouchouicha B. (2025). Experimental characterization and numerical simulation of compressive residual stresses from shot peening in industrial contexts. Trans. Indian Inst. Met..

[11] Balan K. (2020). Are You Still Using MIL-S-13165. The Shot Peener Magazine|Summer.

[12] Mort P., Feltner L. (2022). Characterization of Shot Size and Shape Distribution.

[13] Feltner L., Gruninger M., Canty T., Mort P. (2024). Characterization of Particle Size and Shape Distributions for Shot Peening Media. The Shot Peener Magazine|Spring.

[14] Miao H., Larose S., Perron C., Lévesque M. (2009). On the potential applications of a 3D random finite element model for the simulation of shot peening. Adv. Eng. Softw..

[15] Feltner L., Mort P. (2025). Probabilistic Assessment of Shot Peening Impact Coverage. The Shot Peener Magazine. Spring.

[16] Feltner L., Stumpf D., Mort P. (2025). On the use of the Fourier transform to determine contact curvature distributions in additive manufacturing powders. Powder Technol..

[17] Feltner L., Mort P. (2023). CSEE Project Update: ISP Flowsheet Model v1.

[18] Ghanbari S., Bahr D.F. (2020). Predictions of decreased surface roughness after shot peening using controlled media dimensions. J. Mater. Sci. Technol..

[19] Dassault Systèmes (2019). Abaqus/Explicit User’s Manual, Version 2019.

[20] Wang Z., Shi M., Gan J., Wang X., Yang Y., Ren X. (2021). The effects of shot distance and impact sequence on the residual stress field in shot peening finite element model. Metals.

[21] Tanaka K. (2019). The cos*α* method for X-ray residual stress measurement using two-dimensional detector. Mech. Eng. Rev..

[22] Kohri A., Takaku Y., Nakashiro M. (2016). Comparison of X-ray residual stress measurement values by cos *α* method and sin2 *ψ* method. Residual Stress..

[23] Feltner L., Mort P. (2025). Spectral Fabric of Stochastic Residual Stress Fields. https://www.researchsquare.com/article/rs-7466816/v1.

[24] Abadi M., Barham P., Chen J., Chen Z., Davis A., Dean J., Devin M., Ghemawat S., Irving G., Isard M. (2016). TensorFlow: Large-Scale Machine Learning on Heterogeneous Systems. https://www.tensorflow.org/.

[25] Klump A., Klaus S., Dietrich V., Schulze V. Prediction of Residual Stress States after modified Shot Peening Treatments using Strain-Rate and Temperature-dependent Material Data. Proceedings of the 14th International Conference on Shot Peening.

[26] Wang C., Hu J., Gu Z., Xu Y., Wang X. (2017). Simulation on residual stress of shot peening based on a symmetrical cell model. Chin. J. Mech. Eng..

[27] Rosenfeld A., Pfaltz J.L. (1966). Sequential operations in digital picture processing. J. ACM (JACM).

